# Modern Therapeutics of the Diseases of Children

**Published:** 1885-07

**Authors:** 


MODERN THERAPEUTICS OF THE DISEASES OF CHILDREN, with Observations on the Hygiene of Infancy. By Joseph F. Edwards, M. D., Editor of the *Annals of Hygiene;* Associate Editor of the *Medical and Surgical Reporter;* Author of Bright's Disease and its Treatment; Fellow of the College of Physicians of Philadelphia. Philadelphia: D. G. Brinton, 115 South Seventh street. 1855.

This book contains three hundred and fifty-six pages, presenting in a concise manner the results of the modern application of therapeutics, in its widest sense, to the diseases of children, and giving the details of the treatment of the most eminent specialists in this branch, both American and foreign. The author, or rather editor, as he might be more appropriately styled, has grouped together a series of papers upon the several affections by those of enlarged experience in treating children, and thus affords a satisfactory resumé of the treatment which long experience has proved to be advantageous, and that which recent discovery has introduced with a fair promise of value. He tells us that no similar work of anything like this extent has ever been published, devoted to this particular field, and it is to be hoped that the profession will find in these pages suggestions of practical utility and a trustworthy adviser in combatting disease among children.

The difficulties of diagnosing the diseases of infants are much greater than in those of persons who have reached the years of discretion, as no intelligent explanation can be received from children as to the seat of pain or other discomforts, and consequently the phenomena which characterize their diseases demand close attention from the physician.

There are many things to be thus learned from the mother or nurse of a sick child, which no special investigation of the medical attendant can bring to light, and it devolves upon those who undertake to treat this class of patients to inquire into the whole history of the case, with its surroundings and domestic relations.

There is perhaps no one cause so productive of trouble with children as deficient or vitiated nourishment, and it not infrequently occurs that a child is starving from the small supply or the deleterious quality of the mother's milk, without any suspicion of the impairment in the lacteal secretion. When a wet nurse can be secured of an unexceptionable character, of course, this affords the best substitute for the mother's milk, and in the failure to obtain a healthy woman with an abundant yield of nutritious food for the child, the milk of a selected cow is preferable to all artificial nourishment. In case the pure cow's milk does not agree with the child, owing to the loss of tone in the stomach from deterioration of the food previously, it may be found advantageous to resort to the use of the peptonizing powder with the milk so as to render it acceptable to the weakened digestive organs, as recommended on page 122 of this work.

The numerous formulæ given would have met the wants of the lamented Sims in his early career, and may relieve others in similar straits.

